# Detection of EGFR gene polymorphisms in non-small cell lung cancer Egyptian patients: a case–control study

**DOI:** 10.1186/s41021-023-00289-y

**Published:** 2023-11-27

**Authors:** Omali Y. El-khawaga, Mohammed F. Al-azzawy, Afaf M. ElSaid, Sherif Refaat, Aliaa N. El-Dawa

**Affiliations:** 1 Biochemistry Division, Chemistry Department, Faculty of Science, Mansoura University, Mansoura, 35516 Egypt; 2 Genetic unit, Department of Pediatrics, Faculty of Medicine, Mansoura University, Mansoura, 35516 Egypt; 3 Lecturer of Medical Oncology, Faculty of Medicine, Mansoura University, Mansoura, Egypt

**Keywords:** EGFR gene, Lung carcinoma, Genetic mutation

## Abstract

**Background:**

Non-Small Cell Lung Cancer displays several genetic mutations including epidermal growth factor receptor. This study's objective was to determine if the EGFR exon19 rs121913438 and exon21 rs121434568 variations play a role in NSCLC susceptibility.

**Methods:**

Case–control research was done at the Mansoura university oncology center including 124 NSCLC patients, and 124 healthy volunteers. blood was used to obtain genomic DNA. ARMS-PCR was used to genotype single-nucleotide polymorphisms.

**Results:**

Molecular study for EGFR exon 19 del. showed NSCLC cases were significantly associated with a higher proportion of heterozygous WD, WD + DD dominant genotypes, and mutant D allele, (*p* < 0.05 for each), with a risk to develop NSCLC. also, NSCLC cases were significantly associated with a higher proportion of heterozygous TG, TG + GG dominant genotype, G mutant allele, (*p* < 0.05 for each), with a risk to develop LC (OR > 1 for each). regarding the two EGFR mutations, TTF1 staining was significantly associated with WD + DD genotypes for EGFR exon 19 del But not EGFR exon 21. No substantial differences were found among all studied cases with CK7 or napsin A Tumor cytochemistry.

**Conclusions:**

The WD heterozygous genotype and D allele in exon 19 del. mutation as well as the TG heterozygous and G allele in exon 21 substitution mutation in EGFR gene are strongly associated with the development of advanced-NSCLC in the Egyptians.

## Introduction

The Global Cancer Observatory (GCO) claims that over 2,200,000 new cases of cancer were registered worldwide in 2020, regardless of gender [1]. Lung cancer ranks among the most common cancers diagnosed worldwide. Based on the evolving characteristics of the most commonly diagnosed subtypes, lung cancer was the world's second-most common cancer in 2020, trailing only breast cancer [2]. Lung tumor mortality is greater than for other types of cancer, owing to late-stage detection. The disease's early stage is characterized by either a poor clinical manifestation or the occurrence of unspecific symptomatology. Almost half of those with lung cancer (46%) are diagnosed when the cancer has already metastasized to other areas of the body, which makes the diagnostic procedure difficult and the treatment more difficult [3]. Therefore, there is an urgent need to discover a highly sensitive and specific biomarker in order to diagnose non-small cell lung cancer (NSCLC) patients at an early stage of the disease process. Special attention is paid to genetic mutations as tumor markers, which can be detected in easily accessible biological fluids, because they can be useful for screening and early diagnosis of cancer (even before its clinical manifestation) as well as corroboration of standard diagnostic methods. EGFR-receptor tyrosine kinases (RTK) play an essential role in initiating and triggering signaling events in both NSCLC and SCLC lung cancer subtypes [4]. EGFR is located on chromosome 7 short arm q22 and spans 110 kb of DNA, which is divided into 28 exons [5]. Normal cells have 40,000–100,000 EGFR receptors for each cell, whereas cancer cells exclusively express more than one million receptors for each cell [6]. EGF modulates its own receptor by expanding EGFR RNA expression via ETF (EGFR-specific transcription factor) expression [7]. EGFR genetic mutations occur at point mutation "hotspots" in the extracellular domain, the kinase domain, and the C-terminal tail [8]. Certain cancers appear to prefer specific mutation sites. For illustration, NSCLCs (non-small cell lung cancer) almost always seem to have kinase domain mutations [8, 9]. EGFR is indeed abundantly expressed in 40 to 80% of NSCLC. The vast majority of EGFR mutations or truncations encourage constitutive EGFR activation by sustaining ligand-independent dimerization at ERBB family receptors [10, 11]. The occurrence of gene mutations in NSCLC varies greatly by ethnicity. (EGFR)-accessing mutations are found in 10–20% of Caucasians, with at least 50% of them being NSCLC patients [12–14]. Two mutations, the deletions that were discovered in exon 19, which affect the conserved Long-range epigenetically active (LREA) domain motif, and the single amino acid substitution L858R through exon 21 at codon 858 (Leucine to Arginine; L858R), are referred to as "classical" EGFR mutations since they account for 85 percent of identified gene mutations in NSCLC and confer responsiveness to EGFR kinase inhibitors (EGFRi) [15].

To our knowledge, there is currently no report in Egypt assessing the impact of EGFR classical mutations on advanced NSCLC by case–control research, which is preferable to cross-sectional studies in respect of risk factor evaluation. This observation-based study was conducted in an Egyptian population of NSCLC patients and matched controls to assess the significance of EGFR mutations in susceptibility to NSCLC in consideration of confounding factors such as gender and age differences between healthy and NSCLC patients, as well as immunohistochemical tumor markers and histopathological subtypes between EGFRmut and EGFR^wt^ in NSCLC patients.

## Materials and methods

### Participants

One Hundred Twenty-four patients with primary NSCLC were admitted to Mansoura University Oncology Center from October 2021 to May 2022, together with 124 age- and sex-matched healthy control individuals. In brief, the data obtained from the medical records of each patient included demographics (age and sex). Patients' clinical information was staged at the time of diagnosis following the tumor/node/metastasis staging system of the American Joint Committee on Cancer (AJCC) tumor/node/metastasis staging system. Patients who took part in the study were classified by the WHO [16]. The dispersion of histological subtypes for NSCLC among the 124 patients was classified into 3 main types: adenocarcinomas, large-cell carcinomas, squamous-cell carcinomas, and miscellaneous-type lesions. The Eigh^th^ Version of TNM Staging of Lung Carcinoma used a combined pathological grading system for tumor tissues to classify patients [17]. Matched healthy controls would be recruited according to the absence of both clinical manifestations and a family history suggesting NSCLC cancer. Participants were excluded from other intestinal diseases or other parts of the original tumor. No one in the control group was a smoker or had a history of any interfering disease or chronic use of any drugs.

All patients in this study underwent a comprehensive clinical examination, which included imaging tests using x-rays, magnetic fields or sound waves. TNM, tumor histopathology, stage, and IHC include TTF1, Napsin A, and CK7. In the laboratory, molecular examinations for genetic changes and CEA tumor marker levels were carried out. All patients and control volunteers signed informed written consent forms. The patients' privacy was preserved by assigning code numbers to each of them.2.2. Blood sampling.

Samples were gathered by taking 5 ml of blood from all patients and controls, either preserved on blank tubes to investigate CEA tumor marker levels or on sterile ethylene diamine tetra acetic acid (EDTA) for analysis of EGFR gene polymorphisms. All samples were obtained and then stored at -20 °C prior to the procedure, and they have been left to be put at room temperature to be used in DNA extraction. Then, samples were analyzed by the PCR technique, followed by gel electrophoresis, to detect gene polymorphisms for EGFR.

### DNA extraction and genotyping

The Easy Pure® Genomic DNA Purification Kit (Cat. No. EE101) was used to extract genomic DNA from peripheral blood leukocytes [18]. The purified DNA was used immediately in PCR application. Evaluation of EGFR exon19 (rs121913438) and exon21 (rs121434568) polymorphisms was done using an amplification refractory mutation system-polymerase chain reaction (PCR). The primers to be used are listed in Table 1.

**Table 1:** Primer pairs used for screening of EGFR exon19 rs121913438, exon21 rs121434568 mutation by ARMS-PCR

**Mutation**	**Primer sequence**	**Size (bp)**
**EGFR** **rs121913438** **(Exon 19)**	Common F (FC): 5`- GTAAATCCACCCAGATCACTG -3` Common R (RC): 5`- GTGTCAAGAAACTAGTGCTGGG -3`	444 bp
	FM: 5`- GTTGGCTTTCGGAGATGTTTTGATAG -3` RN: 5`- CCCGTCGCTATCAAGGAATTAA -3`	134 bp
		325 bp
**EGFR** **rs121434568** **(Exon 21)**	FC: 5`—TGACCCTGAATTCGGATGCA -3` RN: 5`- TTCCGCACCCAGCAGTTTGGCTA -3`	199 bp
	FC: 5`—TGACCCTGAATTCGGATGCA -3` RM: 5`- CGCACCCAGCAGTTTGGTT -3`	196 bp

### Ampilification of geomic DNA by ARMS-PCR

The optimization of amplification was performed under the conditions listed in Table 2.

**Table 2:** Optimization of PCR condition for EGFR (exon19) and (exon 21)

**VARIANT NAME**	**Cycle name**	**Temperature °C**	**Time**	**Number of Cycles**
**EGFR** **(Exon 19)** **rs121913438**	Initial denaturation	95	5 min	1
	Denaturation	95	35 s	35
	Annealing	65	35 s	
	Extension	72	40 s	
	Final extension	72	5 min	1
	Soak	4	∞	1
**EGFR** **(Exon 21)** **rs121434568**	Initial denaturation	95	5 min	1
	Denaturation	94.5	40 s	35
	Annealing	61.5	40 s	
	Extension	72	50 s	
	Final extension	72	5 min	1
	Soak	4	∞	1

ARMS-PCR was used for the detection of EGFR exon 19 (rs121913438) and exon 21 (rs121434568) according to the method [19]. Each rs121913438 PCR mixture had a total volume of 30 μl. Each tube contained 6 μl of external primers and 15 µl of master mix, which was mixed well with 3 µl of DNA, 3 µl of forwarding mutant (FM), and reverse normal (RN). The procedure rendered three bands (internal control at 444 bp, WW (wild genotype) at 134 bp and DD (mutant genotype) at 325 bp). For rs121454568, two tubes were used for every subject. Each PCR reaction mixture was performed in an overall volume of 24 µl including 4 µl of forwarding control (FC), 4 µl of reverse normal primer (RN) or 4 µl of reverse mutant primer (RM), and 12 µl of master mix (COSMO PCR RED Master Mix (W10203001), willow fort) in an Eppendorf Gradients Thermal cycle. The procedure rendered two bands (TT (wild genotype) at 199 bp and GG (mutant genotype) at 196 bp). Samples were amplified using a T-professional thermocycler (Biometra, Germany). The PCR products were electrophoresed on a 2.5% agarose gel that was stained with ethidium bromide and visualized under UV light.

### Analysis of CEA serum level

Serum levels of CEA were detected in NSCLC patients using ELISA kits according to the manufacturer's protocols (catalog no. EHCEA; Thermo Fisher Scientific, Inc., Waltham, MA, USA). Finally, the serum concentrations of CEA were measured using an enzyme microplate reader at 450 nm.

### Statistical analysis

The data collected were analyzed and lobulated using the SPSS software package (IBM Corp., 2017. windows SPSS Statistics, Version 25.0. Armonk, NY: IBM Corp.). In terms of the research population's demographic and clinical features, categorical variables such as gender are reported as frequencies with percentages. 95% confidence interval and odd ratio (OR) conferred by potential correlations regarding the EGFR gene polymorphisms with the risk and progression of NSCLC The probability level (P) of less than 0.05 was defined as a criterion of significance. Hardy-Weinberg equilibrium (HWE) for the two different SNPs was calculated by goodness-of-fit between the observed and expected genotype frequencies.

## Results

The current study represents an observational study of 124 control volunteers and124 non-relevant patients with NSCLC. The Hardy-Weinberg equation revealed that all studied genotypes in the control group as well as in NSCLC cases were in HW equilibrium, as no significant differences were found between observed and expected counts in each group. Patients included 74 males representing the majority of cases and 50 females. The patients ‘ages ranged with a mean (± SD) of 56.0 (± 11.5) years. The control volunteers were 124 healthy individuals including 78 males and 46 females. The controls` mean age (± SD) was 55.4 (± 11.0) years. As shown in Table 3, both patients and healthy controls appeared to be matched in terms of age (*P*=0.272) and gender (*P*=0.602).

**Table 3:** Comparison of age and gender among studied groups

	**Control** ***n* = 124**		**Cases** ***n* = 124**		***P***
**Age (years) mean ± SD**	55.4	11.0	56.0	11.5	0.272
**Males n (%)**	78	62.9%	74	59.7%	0.602
**Females n (%)**	46	37.1%	50	40.3%	

*SD* standard deviation; Numerical data are expressed as mean and SD

Among the 124 patients studied, 98 (79.0%) had adenocarcinomas, 12 (9.7%) had large cell carcinomas, 8 (6.5%) had squamous cell carcinomas, and 6 (4.8%) had other subtypes of NSCLC. According to the stage grade, 77 patients (64.2%) were in the third grade, 41 (34.2%) were in the second grade, and only 2 (1.79%) were in the first grade. According to the Eighth Edition of TNM Staging of Lung Cancer [16], patients were classified into a combined pathological system for tumor tissues, into stages I, II, III, and IV. 94 cases (75.8%) were in the IV stage, while 25 patients (20.2%) were in the III stage. The percentage of positive TTF1 staining was 51.6%, CK7 was positive at 71.0%, and napsin A was positive at 33.9%.

### Distribution of EGFR rs121913438 gene polymorphism in Controls compared to NSCLC patients

**Fig. 1:**
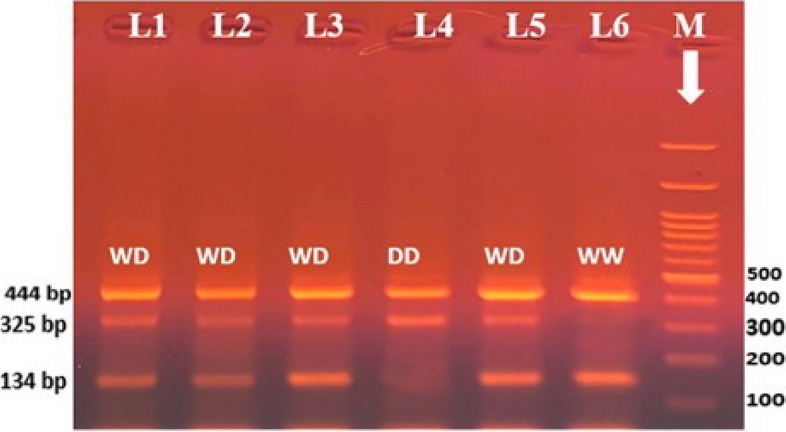
Tetra-ARMS PCR electrophoretic pattern of the EGFR (rs121913438) product, where each lane represents one participant. M stands for DNA marker (100 bp). The internal control is shown by the 444 bp band. Based on the primer, specific 325 bp bands represent the mutant (D) allele, and specific 134 bp bands represent the wild (W) allele. WD heterozygous is represented by lanes 1, 2, 3, and 5. Lane 4 indicates mutant homozygous, where the W allele is absent from the lane and the D allele is present at 325 bp. Lane 6 represents wild-type homozygosity, with the W allele appearing at 134 bp and the D allele absent

The genotyping study was performed on 124 patients with NSCLC and 124 healthy controls. Both genotype distributions of the rs121913438 of EGFR in the two groups were in Hardy-Weinberg equilibrium. As shown in Table 4, a deviation in genotype distribution was observed for EGFR genotype polymorphisms when comparing NSCLC patients and healthy volunteers. Concerning the rs121913438 polymorphic genotype, the WD heterozygous genotype was found in 43 NSCLC patients (34.7%), which is more than the 22 (17.7%) controls. The DD homozygous genotype was observed in only 2 (1.6%) of the NSCLC patients and none of the healthy controls. while the WW homozygous genotype was lower in patient 79 (63.7%) than in the control 102 (82.3%). The WD genotype increased the chance of NSCLC (odds ratio [OR], 1.794; confidence interval [CI], 95% (1.246–2.583); probability (*P* = 0.002)) compared to the WW genotype (wild type). In comparison to the W allele (wild type), the D allele, which represents 47 (19%) of NSCLC and 22 (8.9%) of healthy volunteers, was linked with a higher incidence of NSCLC (OR, 1.764; [CI] 95%, (1.239–2.510); *P* = 0.002) (Fig. 1).

**Table 4:** Association of EGFR rs121913438 genotype and alleles with risk of NSCLC susceptibility

**EGFR exon 19 del**		**Control** ***n* = 124**		**NSCLC patients** ***n* = 124**		*P*	**OR**	**(95% CI)**
		**N**	**%**	**n**	**%**			
**Genotype**	**WW**	102	82.3	79	63.7	Wild type		
	**WD**	22	17.7	43	34.7	0.002	1.794	1.246–2.583
	**DD**	0	0	2	1.6	1		
	**WD + DD**	11	17.7	45	36.3	0.001	1.830	1.275–2.626
**Allele**	**W**	226	91.1	201	81	Wild type		
	**D**	22	8.9	47	19	0.002	1.764	1.239–2.510

*P* probability

*p* < 0.05 is significant; Odds ratio [OR]; Confidence interval [CI], Logistic regression analysis was used

### Distribution of EGFR (exon 21) rs121434568 gene polymorphism in Controls compared to NSCLC patients:

**Fig. 2:**
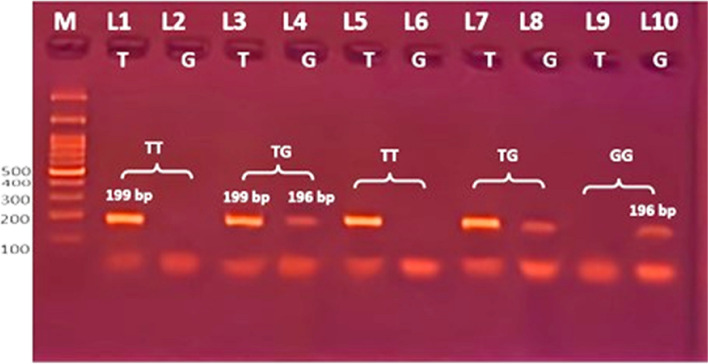
Individual ARMS PCR electrophoretic pattern of the EGFR gene (rs121434568) product, with each lane representing a different participant. M stands for DNA marker (100 bp). Depending on the primer, specific 199 bp bands represent the T allele, and specific 196 bp bands represent the G allele. The TG heterozygous genotyping is represented by lanes 3, 4, 7, and 8. where lanes 3 and 7 represent the T allele band and lanes 4 and 8 represent the G allele. Lanes 1, 2, 5, and 6 indicate TT homozygosity; whereas the G allele is absent from lanes 2 and 6, the T allele is present on lanes 1 and 5. Lanes 9 and 10 are GG homozygous, with the G allele appearing at 196 bp on lane 10 and the T allele absent from lane 9

In both NSCLC and healthy control groups, the genotype distribution of the rs121434568 polymorphism of EGFR was in Hardy-Weinberg equilibrium. As shown in Table 5, a statistically significant difference was found between NSCLC and controls. The TG heterozygous genotype was identified in 38 NSCLC patients (30.6%), which was higher than in 15 healthy controls (12.1%). Also, the GG homozygous genotype was found in NSCLC patients with 5 (4%) but not in the control group. while the TT homozygous genotype was lower in 81 patients (65.3%) than in 109 control subjects (87.9%). The TG genotype in patients shows a highly significant difference from controls (odds ratio [OR], 2.104; [CI], 95% (1.406-3.147); *P* < 0.001), increasing the risk of NSCLC in comparison with the TT genotype (wild type). The G allele, which represents 48 (19.4%) of NSCLC cases and 15 (6.0%) of controls, was associated with an increased risk of NSCLC (OR, 2.125; [CI] (1.460-3.091); *P* < 0.001) when compared to the T allele (wild type) (Fig. 2).

**Table 5:** Association of EGFR rs121434568 genotype and alleles with risk of NSCLC susceptibility

**EGFR** **exon 21** **(T > G)**		**Control** ***n* = 124**		**NSCLC patient** ***n* = 124**		*P*	**OR**	**(95% CI)**
		**n**	**%**	**n**	**%**			
**Genotype**	**TT**	**109**	**87.9**	81	65.3	Wild type		
	**TG**	15	12.1	38	30.6	< 0.001	2.104	(1.406 -3.147)
	**GG**	0	0	5	4	1		
	**TG + GG**	15	12.1	43	31	< 0.001	2.301	(1.555–3.405)
**Allele**	**T**	233	94.0	200	80.6	Wild type		
	**G**	15	6.0	48	19.4	< 0.001	2.125	(1.460 -3.091)

*P* probability

*p* < 0.05 is significant; Odds ratio [OR]; Confidence interval [CI], Logistic regression analysis was used

### Association of EGFR genotype polymorphisms with other parameters

Numerous variables were investigated with regard to the EGFR mutation studied. As shown in Table 6, the overall frequency of the EGFR exon 19 deletion mutation found in adenocarcinomas was 84.4% (38 of 45), followed by 13.3% (6 of 45) of large cell lung cancer and 2.2% (1 of 45) of squamous cell lung cancer. Two-sided χ tests revealed no significant association between the histology subtype (*p* = 0.081) or grade (*p* = 0.456) and the existence of the EGFR exon 19 mutations. With respect to the TNM staging of all NSCLC patients, there was no relation between EGFR exon 19 rs121913438 and stage (*p* = 0.150). The tumor cytochemistry markers TTF1, CK7, and napsin A were investigated in the studied patient cases. TTF1 staining was significantly associated with WD+DD genotypes (*p* = 0.015) in comparison to WW genotyping. while CK7 and napsin A were found to have no significant association with this variant.

**Table 6:** Association of EGFR exon 19 del with studied parameter

**EGFR exon 19** **del rs121913438**		**Wild genotype**		**Mutant genotype**		*P*
		**(WW)**		**WD + DD**		
		**n**	**%**	**n**	**%**	
**Pathology**	**adenocarcinoma**	60	75.9%	38	84.4%	0.081
	**large cell carcinoma**	6	7.6%	6	13.3%	
	**squamous cell**	7	8.9%	1	2.2%	
	**Others**	6	7.6%	0	0.0%	
**Grade**	**1**	1	1.3%	1	2.2%	0.456
	**2**	23	30.7%	18	40.0%	
	**3**	51	68.0%	26	57.8%	
**Stage**	**I**	2	2.5%	0	0.0%	0.150
	**II**	2	2.5%	1	2.2%	
	**III**	20	25.3%	5	11.1%	
	**IV**	55	69.6%	39	86.7%	
**Tumor** **cytochemistry**	**TTF1**	34	43.0%	30	66.7%	0.015
	**CK7**	53	67.1%	35	77.8%	0.403
	**napsin A**	27	34.2%	15	33.3%	0.874

*P* probability

*p* < 0.05 is significant

As shown in Table 7, the EGFR (exon 21) rs121434568 polymorphism yields different results for histopathological classification. The overall frequency of the exon 21 mutation was found in 88.4% (38 of 43) of adenocarcinomas and 11.6% (5 of 43) of large-cell lung cancer. The TT genotype was significantly associated with squamous cell carcinoma (*p* = 0.049) when compared to the TG + GG genotype. Otherwise, no significant association was found regarding EGFR exon 21 and tumor pathology among all studied cases. There was no significant association between tissue grade and tumor stage (*p* > 0.05). No significant relationship was found between the tumor cytochemistry (TTF1, CK7, and napsin A) and rs121434568.

**Table 7:** Association of EGFR exon 21 del with studied parameter

**EGFR (exon 21)** **rs121434568**		**Wild genotype**		**Mutant genotype**		*P*
		**TT**		**TG + GG**		
		**n**	**%**	**n**	**%**	
**Pathology**	**Adenocarcinoma**	60	74.1%	38	88.4%	0.063
	**large cell carcinoma**	7	8.6%	5	11.6%	0.751
	**squamous cell**	8	9.9%	0	0.0%	**0.049**
	**Others**	6	7.4%	0	0.0%	0.092
**Grade**	**1**	0	0.0%	2	4.7%	0.241
	**2**	27	35.1%	14	32.6%	
	**3**	50	64.9%	27	62.8%	
**Stage**	**I**	2	2.5%	0	0.0%	0.448
	**II**	2	2.5%	1	2.3%	
	**III**	19	23.5%	6	14.0%	
	**IV**	58	71.6%	36	83.7%	
**Tumor** **cytochemistry**	**TTF1**	37	45.7%	27	62.8%	0.070
	**CK7**	54	66.7%	34	79.1%	0.148
	**napsin A**	25	30.9%	17	39.5%	0.332

The tumor marker CEA level was investigated for all patients. As shown in Table 8, no significant association was found regarding EGFR exon 19 del. (*p* = 0.423) or exon 21 (*p* = 0.863) with CEA level among all studied cases.

**Table 8:** Association of CEA level with EGFR exon 19 del and exon 21 with among all studied cases

**CEA ng/ml**		**Median**	**range**	*p*
**EGFR exon 19**	**WW**	6 ng/ml	1–1500	0.423
	**WD + DD**	7 ng/ml	1–217	
**EGFR exon 21**	**TT**	6 ng/ml	1–1500	0.863
	**TG + GG**	6 ng/ml	1–239	

## Discussion

Numerous human cancers expressed EGFR, which has been linked to tumor progression and grade, as well as a poor cancer prognosis [20]. The EGFR signaling pathway was activated in more than half of NSCLC patients and was important in cancer cell proliferation and invasion [21].

This study for genetic diagnosis is based on an association study using polymorphic markers to detect carriers among unrelated patients and control subjects for exon 19 of EGFR and the mutation of exon 21 (L858R), which are the most common EGFR mutations. In the current investigation, we examined the association between two SNPs in the EGFR gene (rs121913438 and rs121434568) and NSCLC risk. Regarding EGFR exon 19 in-frame deletion, WW was considered the reference genotype, and W was considered the ancestral allele. From the result, we found that LC cases were significantly associated with a higher proportion of WD, DD genotype, and D allele (*P* <0.05 for each) and an increased risk of developing LC (OR > 1 for each). Regarding the EGFR exon 21 missense variant, TT was considered the reference genotype, and T was considered the ancestral allele. If T>G conversion occurs in the population, there will be a highly significant (*P* < 0.001) relationship between GG, TG genotype, G allele, and NSCLC, with an odd ratio greater than 1. The present case-control research investigates that the EGFR deletion mutation of exon 19 and the point mutation of exon 21 are disproportionately distributed between NSCLC and controls in Egyptians. So, there has been a powerful link between EGFR gene classical mutations and the chance to get NSCLC. To understand the pro-carcinogenic role of rs121434568 and rs121913438 SNPs, it is valuable to decipher the EGFR role in the issue of cancer. In this regard, Structural studies have revealed that L858R and exon 19Del. destabilize the inactive conformation by sustaining ligand-independent dimerization to ERBB family receptors, even in absence of EGF, leading to increased receptor dimerization and promote a constitutively activated form of EGFR, compared to EGFR^WT^ [22, 23]. To our knowledge, this is the first case-control genotype correlation of those variants in Egyptian that can be used as single-nucleotide biomarkers in genetic analysis to help predict disease.

The incidence of EGFR mutations in NSCLC is dependent on tumor type and ethnic background [10]. From previous studies in Japan, it is frequently seen, especially in adenocarcinomas [24]. These results are inconsistent with our findings, as there was no association between the EGFR exon 19 deletion mutation and any NSCLC histopathology subtype. But squamous cell carcinoma cases showed no mutation for the EGFR exon 21 genotype. The last point of view coincides with squamous carcinoma genomic atlas which shows that EGFR is seldom mutated SQC in lung [25]. Another previous study in the Lebanese population demonstrated a significant correlation between EGFR mutations and well-differentiated tumor pathology [26]. An increase in the incidence of adenocarcinoma has been observed in Japan. Considering the higher probability of EGFR mutation in the Japanese population, one may assume that the overall incidence of EGFR^mut^ in NSCLC is also increasing [27].

TTF1 is a marker currently used in routine clinical practice to distinguish lung cancer metastases [28]. TTF1's role in lung pathology and differential diagnosis is well documented because it primarily exists in enhanced surface-active substances and Clara cell secretory protein region binding sites that can maintain lung cancer cell activity [29], but its prognostic value, particularly for patients with advanced-stage lung cancer with an EGFR genetic mutation, has received less attention. The percentage of positive TTF1 expression reported in our study (51.6%) was found to be significant with the EGFR exon 19 deletion. This result is in accordance with previously reported studies [28, 30]. Earlier, Sun et al. [29] reported that TTF-1 had a strong correlation with EGFR mutations.

According to research papers, serum tumor markers (STMs) could indeed assist in the detection of clinically suspected cancer as well as cancer with an unknown primary site. STMs, such as carcinoembryonic antigen (CEA), are routinely employed for NSCLC screening and recurrence evaluation and have been linked to prognostic variables such as a higher TNM stage [31]. These findings raise the chance of a link between CEA levels and EGFR genetic variations. According to Sordella et al., the mutated EGFR gene can abnormally power up the downstream signal transduction pathway and further encourage transcription factor expression and activation, establishing the antiapoptotic pathway and accelerating cell proliferation, both of which have significant implications in lung tumorigenesis [32]. According to Jin et al., the EGFR mutation rate increased with CEA serum levels in Chinese patients with ADC [33]. However, in all cases studied, there was no significant relationship between EGFR exon 19 or exon 21 genetic variants and CEA levels.

In conclusion, our results Confirm that the EGFR deletion mutation in exon 19 and T > G substitution mutation in exon 21 polymorphisms are associated with susceptibility to advanced NSCLC in the Egyptians. Upcoming research utilizing a larger sample size of individuals and the study of the whole genome sequence are critical requirements for more accurate diagnosis of mutations and essential for therapeutic considerations and genetic guidance that contribute to a better chance of survival in the Egyptian population.

## Data Availability

The data provided in this study are accessible upon request from the corresponding author. Because of local law restrictions, the data is not publicly available.
